# Activation of *Wnt* signaling promotes hippocampal neurogenesis in experimental autoimmune encephalomyelitis

**DOI:** 10.1186/s13024-016-0117-0

**Published:** 2016-07-14

**Authors:** Reiner Schneider, Barbara Koop, Friederike Schröter, Jason Cline, Jens Ingwersen, Carsten Berndt, Hans-Peter Hartung, Orhan Aktas, Tim Prozorovski

**Affiliations:** 1 Department of Neurology, Medical Faculty, Heinrich-Heine-University Düsseldorf, Merowingerplatz 1a, Moorenstr.5, 40225 Düsseldorf, Germany; 2 Present address: Institute for Stem Cell Research and Regenerative Medicine, Medical Faculty, Heinrich-Heine-University Düsseldorf, Moorenstr. 5, 40225 Düsseldorf, Germany

**Keywords:** *Wnt*, Hippocampus, Neurogenesis, EAE, Multiple sclerosis

## Abstract

**Background:**

Disease progression in multiple sclerosis (MS) and experimental autoimmune encephalomyelitis (EAE), as one of its animal models, is characterized by demyelination and neuronal damage in white and gray matter structures, including the hippocampus. It is thought that dysfunction of the hippocampus, a primary locus of learning and memory consolidation, may contribute to cognitive impairment in MS patients. Previously, we reported an increased generation of hippocampal neuronal progenitors in the acute stage of EAE, whereas the microenvironmental signals triggering this process remained uninvestigated.

**Results:**

In the present study, we used the *Wnt* signaling reporter mouse Axin2^LacZ^, to elucidate the molecular mechanisms underlying the activation of the hippocampal neurogenic niche upon autoimmune neuroinflammation. Histological and enzymatic examinations of β-gal during the disease course of EAE, allowed us to survey hippocampal *Wnt*/β-catenin activity, one of the key signaling pathways of adult neurogenesis. We found that *Wnt* signaling is transiently upregulated in the acute stage of disease, consistent with a timely induction of canonical *Wnt* ligands. The enhancement of signaling coincided with hippocampal neuronal damage and local expression of immune cytokines such as TNFα and IFNγ, implicating the role of the inflammatory milieu in activation of the *Wnt*/β-catenin pathway. Supporting this finding, we show that transient exposure to pro-inflammatory cytokine TNFα triggers *Wnt* signaling in hippocampal organotypic slice cultures. Importantly, inflammation-mediated activation of the *Wnt*/β-catenin pathway was associated with enhanced neurogenesis in vitro and in vivo, indicating its potential role in hippocampal tissue regeneration and repair.

**Conclusions:**

This study raises the possibility that enhancement of *Wnt* signaling may support neurogenic processes to cope with neuronal deficits upon immune-mediated neuroinflammation.

**Electronic supplementary material:**

The online version of this article (doi:10.1186/s13024-016-0117-0) contains supplementary material, which is available to authorized users.

## Background

A large body of research highlights the involvement of hippocampal pathology in cognitive disability that is commonly observed in patients with multiple sclerosis (MS), an inflammatory demyelinating and neurodegenerative disease of the central nervous system (CNS) [1, 2]. Structural changes in the hippocampus were shown to be associated with MS-related memory impairment [3] and may occur prior to cognitive dysfunction [4, 5], suggesting a therapeutic opportunity for reversing memory decline in MS patients (reviewed in [5–7]). Examinations of neurobiological alterations in post-mortem brain tissue indicate frequent and extensive hippocampal demyelination [8] resulting in alterations of synaptic function, axonal transport [9] and neuronal loss in the Ammon’s horn 1–3 (CA1-3) regions [9, 10]. Furthermore, axonal demyelination was shown to be accompanied by drastic changes in the expression of specific neuronal genes [9] and neuronal miRNA [11] suggesting disturbance of molecular pathways involved in physiological homeostasis of hippocampal tissue. Experimental autoimmune encephalomyelitis (EAE), an animal model of MS, mimics many aspects of hippocampal pathology and its clinical expression in the human disease such as demyelination, alteration in synaptic plasticity and transmission [12, 13], neuronal loss [12], cognitive deficits [14] and thus, becomes an important model for the characterization of mechanisms involved in hippocampal neuropathology and MS-related memory deficits. Our previous data identified adult hippocampal neurogenesis, an important player in cognition [15], as a primary target of autoimmune-mediated neuroinflammation [16]. The analysis of hippocampal gene expression in acute and chronic stage of EAE revealed alterations in the transcriptional profile of genes relevant for key neurogenic pathways in the adult brain, including those of *Wnt* signaling [16]. Considering the potential role of the *Wnt* pathway in tissue renewal and regeneration [17, 18], we here examined the impact of autoimmune neuroinflammation on hippocampal *Wnt*/β-catenin activity by using Axin2^lacZ/+^ reporter mice. We found that T cell-mediated autoimmune neuroinflammation leads to hippocampal neuronal damage occurring early in disease progression and may potentially trigger the activation of *Wnt* signaling via upregulation of a specific set of *Wnt* ligands. Transient increase in the *Wnt* activity in the acute phase was associated with increased proliferation and generation of doublecortin-positive (Dcx^+^) neuronal precursor cells. Further, we identified the inflammatory mediator tumor necrosis factor alpha (TNFα) as a potent enhancer of *Wnt* signaling activity and subsequently, the proliferation of progenitor cells in the hippocampal neurogenic niche.

## Results

### Increased *Wnt* activity in the acute phase of EAE

**Fig. 1:**
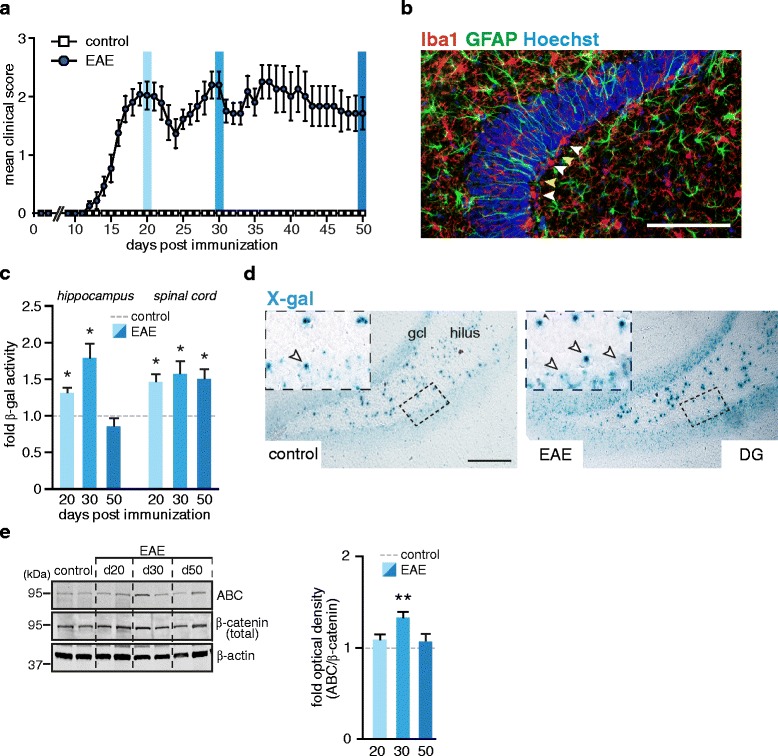
Analysis of *Wnt* activity in EAE. **a** Disease course in Axin2^lacZ/+^ mice with chronic EAE. EAE was induced by immunization with MOG_35–55_. Control group (CFA) were injected with an adjuvant cocktail without antigen. Data are shown as a mean clinical score ± SEM. Blue columns indicate the time points when mice were sacrificed for analysis: days 20, 30 and 50 post immunization. **b** Representative image of Iba1, GFAP co-immunostaining in hippocampal section of an EAE mouse at day 30. White arrow heads indicate reactive microglia Iba1^+^ (red) located in close proximity to radially oriented GFAP^+^ cells (green; yellow arrow heads) resembling radial glia progenitor cells of the SGZ. Nuclei were counterstained with Hoechst (blue). Scale bar: 100 μm. **c** β-gal assay of hippocampal and spinal cord tissue. Histogram represents mean + SEM of fold-changes of β-gal activity in EAE mice relative to respective controls (CFA) set as 1. Day 20 (EAE, *n* = 6; CFA, *n* = 3); day 30 (EAE, *n* = 4; CFA, *n* = 3); day 50 (EAE, n = 8; CFA, *n* = 4). Two-tailed, unpaired Student’s *t*-test, *p < 0.05. **d** Upregulation of *Axin2* expression in the DG of EAE mice. Expression of *lacZ* and thus activation of *Axin2* promoter were detected by X-gal staining on hippocampal sections from EAE mice (day 20) and respective controls (CFA). Representative images are shown. Top insets show magnified cells of the outlined SGZ region. Arrow heads mark X-gal positive cells. Gcl, granular cell layer. Scale bar, 100 μm. **e** Immunoblot analysis of β-catenin in the hippocampus of EAE and control (CFA) animals. Hippocampal proteins were extracted and processed for Western blotting with antibodies against active β-catenin (ABC) and total β-catenin. β-actin was probed for analysis of protein loading. Right histogram represents mean + SEM of fold-changes of normalized optical densities in EAE mice relative to controls (CFA) set as 1. Day 20 (*n* = 6); day 30 (*n* = 4); day 50 (*n* = 6) and control CFA mice (*n* = 6). Two-tailed, unpaired Student’s *t*-test, *p < 0.05 and ** *p* < 0.01

In the adult brain, *Wnt* signaling pathway is persistently active in the hippocampus and is of high importance for homeostatic neurogenesis [19]. To evaluate the impact of autoimmune-mediated neuroinflammation on hippocampal *Wnt*/β-catenin signaling we induced EAE in Axin2^lacZ/+^ reporter mice by immunization with an encephalitogenic CNS self-antigen, myelin oligodendrocyte glycoprotein (MOG_35–55_) peptide. Like other feedback inhibitors in various pathways, *Axin2* is induced in response to the *Wnt* signaling cascade and is widely used as readout of *Wnt*/β-catenin activity. Mutant mice, lacking one functional *Axin2* allele, are born normal and upon immunization developed typical non-remitting chronic EAE with a disease onset at 11–12 days post immunization (d.p.i.). (Fig. 1a). Histological examinations revealed the occurrence of typical perivascular infiltrates, predominantly in the white matter of the spinal cord (Additional file 1: Figure S1a-b). These infiltrates were characterized by increased Iba1 immunoreactivity (the marker for microglia and macrophages). Interestingly, microglia activation was evident in the hippocampus and particularly in the hippocampal subgranular zone (SGZ). In this region, microglia were localized in close proximity to SOX2/GFAP-double positive cells with prototypical morphology of radial glia-like cells (RGCs or type-1 cell) and horizontal progenitors, both the primary stem cells of the adult dentate gyrus (DG) (Fig. 1b and Additional file 1: Figure S1d) [20, 21]. Despite the rare occurrence, we also found perivascular lesions enriched with Iba1 immunoreactive cells located in close proximity to the SGZ, the germinative zone of the hippocampal DG (Additional file 1: Figure S1c). Analysis of β-galactosidase (β-gal) activity in Axin2^lacZ/+^ mice showed the enhancement of *Wnt* signaling in the hippocampal tissue at days 20 and 30 after disease induction (1.31–1.78 fold), while at day 50 its activity resumed to the levels observed in control CFA/PTX-immunized mice (Fig. 1c). In the spinal cord, increased *Wnt* signaling was observed in all disease stages examined (Fig. 1c), whereas no significant changes were detected in the frontal/motor cortex (Additional file 1: Figure S1e), the area that was virtually not affected by inflammation. Only modest increase in β-gal activity was observed in the cerebellum (Additional file 1: Figure S1f). Of note, only trace amounts of β-gal activity were detected in naive or EAE tissue from wt (Axin2^+/+^) mice (data not shown), proving the accuracy of the β-gal measurements and the minor impact of senescence-associated processes. Additionally, we confirmed the results of the β-gal assay in the spinal cord and different parts of the brain by qPCR analysis of the *Axin2* gene expression in the acute stage of EAE (Additional file 1: Figure S1g). The distribution pattern of *LacZ* expression in the DG examined by X-gal staining revealed increased signal intensity mostly in cells of the granular layer including the SGZ and in some cells of the hilus (Fig. 1d). β-gal activity in the hippocampal and spinal cord tissues positively correlated with clinical disease score in individual mice (Additional file 1: Figure S1.h, Pearson correlation, *r* = 0.58; *p* = 0.018 and *r* = 0.52; *p* = 0.036, respectively). Taken together, our results revealed a brain area-specific and disease severity-dependent upregulation of *Wnt* signaling in EAE mice.

Next, we examined the activation state of *Wnt* signaling mediators in naive and EAE mice. The ligation of *Wnts* inducing the canonical pathway leads to stabilization (de-phosphorylation) of β-catenin via inactivation (phosphorylation at Ser9) of GSK-3β, a member of the β-catenin destructive complex. Supporting the results of the enzymatic assay, we found a significant increase in active β-catenin (ABC)/total β-catenin ratio at day 30 of EAE mice in comparison to their respective controls (Fig. 1e). Taken together, our results indicate a transient upregulation of hippocampal *Wnt* signaling in acute stages of EAE (20–30 d.p.i.). In contrast to this, sustained increase in *Wnt* activity was observed in the spinal cord through all EAE stages examined (Fig. 1c), which was correlated with a large number of inflammatory foci (Additional file 1: Figure S1a-b).

### Induction of *Wnt* ligands in passive EAE

**Fig. 2:**
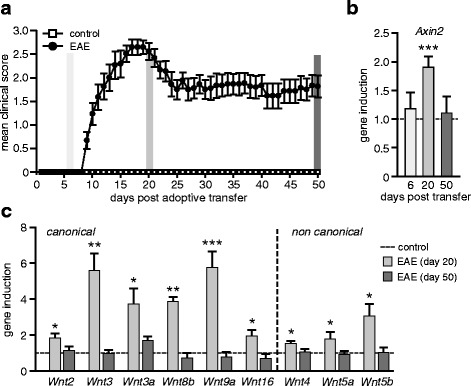
Gene expression analysis of *Wnt*s in the hippocampus of EAE mice. **a** Disease course in passively transferred EAE. Female SJL/J mice received activated encephalitogenic T lymphocytes derived from immunized donors and re-stimulated with encephalitogenic PLP_139–151_ peptide. Control mice were injected with PBS. Data are shown as mean clinical score ± SEM. Grey columns indicate the time points when mice were sacrificed for analysis: days 6, 20 and 50 after adoptive transfer. **b** qPCR analysis of *Axin2* gene expression in the hippocampus of mice with passively transferred EAE. Data represent mean of fold-changes + SEM of gene expression in EAE mice relative to respective controls set as 1. Expression of *Axin2* was normalized to *Gapdh*. Day 6 (EAE, *n* = 5; control, *n* = 4); day 20 (EAE, *n* = 6; control, *n* = 8); day 50 (EAE, *n* = 12; control, n = 11). Two-tailed, unpaired Student’s *t*-test, *** *p* < 0.001. **c** qPCR analysis of selected *Wnts* genes in the hippocampus at early (day 20) and chronic (day 50) stages of passive EAE. Histogram represents mean + SEM of fold-changes relative to control group set as 1. Gene expression was normalized to *Gapdh.* Day 20 (EAE, *n* = 4–6; control, *n* = 4); day 50 (EAE, *n* = 8–13; control, *n* = 7–11). Two-tailed, unpaired Student’s *t*-test, * *p* < 0.05, ** *p* < 0.01 and *** *p* < 0.001

The *Wnt* gene family encodes for 19 cysteine-rich secreted signaling molecules. To determine the particular *Wnt* ligands activated in the hippocampi of EAE mice, we performed *Wnt* gene expression profiling analysis. To exclude possible confounders associated with an active immunization process, this examination was performed in the passive model of EAE induced by adoptive transfer of myelin specific T cells (Fig. 2a). As a first result, we noticed a transient upregulation of hippocampal *Axin2* on the transcriptional level (Fig. 2b). This observation extends our findings from actively immunized Axin2^lacZ/+^ mice and is indicative of enhanced *Wnt* signaling in the acute stage of disease independent from immunization process. Notably, induction of *Axin2* messenger RNA (mRNA) expression has occurred neither prior to the onset of disease (day six after adoptive transfer) nor in the chronic stage (day 50) (Fig. 2b). At day 20, EAE mice exhibited higher mRNA levels of a number of *Wnt* genes relevant for the canonical β-catenin/TCF pathway (*Wnt2*, *Wnt3*, *Wnt3a*, *Wnt8b*, *Wnt9a* and *Wnt16*; fold inductions ranging from 2 to 6) and those signaling through β-catenin-independent mechanisms (*Wnt4*, *Wnt5a*, *Wnt5b*; fold inductions ranging from 1.5 to 3.5) (Fig. 2c). Other *Wnt* ligands found to be expressed in the hippocampus (*Wnt7a*, *Wnt7b*, *Wnt10a*, *Wnt11*) exhibited no significant differences compared to controls (Additional file 2: Figure S2a). We also elucidated the gene expression profile of up-regulated *Wnt* ligands in other CNS regions of EAE mice. In the acute stage, we found an induction of *Wnt2*, *Wnt3*, *Wnt5a*, *Wnt5b* in the spinal cord tissue and *Wnt5a* in the cerebellum (Additional file 2: Figure S2b). As it was observed in actively-immunized mice (Fig. 1c), the transcriptional levels of *Wnts* in the chronic stage of passive EAE returned to the levels detected in control animals (injected with PBS) confirming the transient profile of enhanced *Wnt* signaling (Fig. 2b, c). Consistent with previous reports in other CNS regions [22, 23], our data revealed that the acute phase of EAE is associated with the induction of several *Wnt* ligands in the hippocampus.

### Upregulation of *Wnt* activity correlated with neuronal injury and inflammatory processes

**Fig. 3:**
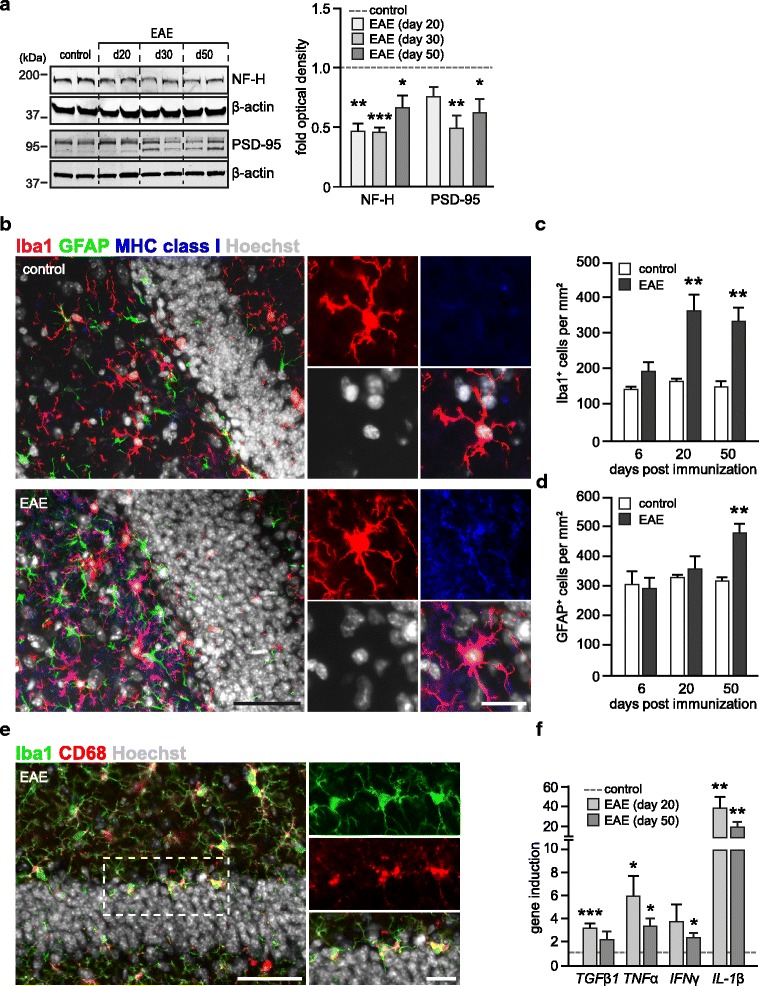
Neuronal damage and neuroinflammation in the hippocampus of EAE mice. **a** Immunoblot analysis of neuronal proteins in the hippocampus of Axin2^lacZ/+^ EAE and control (CFA) animals. Hippocampal proteins were extracted and processed for Western blotting with antibodies against neurofilament-H (NF-H) and postsynaptic protein 95 (PSD-95). β-actin was probed for analysis of protein loading. Right histogram represents mean + SEM of fold-changes of normalized optical densities in EAE mice relative to controls (CFA) set as 1. Day 20 (*n* = 6); day 30 (*n* = 4); day 50 (*n* = 6) and control CFA mice (*n* = 6). Two-tailed, unpaired Student’s *t*-test, *p < 0.05 and ** *p* < 0.01 **b** Representative image of Iba1, GFAP and MHC class I co-immunostaining in a hippocampal section of an EAE mouse and a healthy control at day 20. The insets on the right show magnified Iba1^+^ macrophage/microglia cells (red) co-labelled with MHC class I (blue). Nuclei were counterstained with Hoechst (grey). Scale bar: 50 μm and 10 μm. **c**-**d** Quantification of Iba1^+^ (c) and GFAP+ (d) cells in the DG of EAE (*n* = 4) and control (*n* = 4) mice. Data represents mean + SEM. Two-tailed, unpaired Student’s *t*-test, * *p* < 0.05. **e** Representative image of Iba1, CD68 co-immunostaining in the DG of EAE mouse at day 20. The insets on the right show magnified microglia cells (green) co-labelled with the activation marker CD68 (red). Nuclei were counterstained with Hoechst (grey). Scale bar: 50 μm and 10 μm. **f** qPCR analysis of gene expression of inflammatory cytokines in the hippocampus at early (day 20) and chronic (day 50) stages of EAE. Histogram represents mean + SEM of fold-changes relative to control (CFA) group set as 1. Expression was normalized to *Gapdh.* Day 20 (EAE, *n* = 3–6; control, *n* = 3–8); day 50 (EAE, *n* = 9–10; control, *n* = 6–7). Two-tailed, unpaired Student’s *t*-test, * *p* < 0.05, ** *p* < 0.01 and *** *p* < 0.001

Upregulation of *Wnt* signaling pathway members was observed in early stages of various pathologies associated with inflammation-mediated tissue injury [17, 18]. To take a closer look at hippocampal neuronal injury in early and late stages of EAE, we analyzed the expression of the axonal marker neurofilament-H (NF-H) and the postsynaptic density protein-95 (PSD-95). The protein levels of PSD-95 were diminished throughout all stages of disease, reflecting the reduction in the number of hippocampal synapses (Fig. 3a). We also found a persistently decreased protein level of NF-H (Fig. 3a), indicating that early neuronal injury is not compensated for in the chronic stage of non-remitting EAE (Fig. 1a). Early neuronal damage was associated with active inflammatory processes, as suggested by an elevated number of Iba1-positive cells (Fig. 3b-c), immunoreactivity for activation markers MHC class I (Fig. 3b), CD68 (Fig. 3e) and significant upregulation of gene transcription of inflammatory mediators (TNFα, IFNγ and interleukin-1 beta (IL-1β)) (Fig. 3f). Of note, strong positive correlation was observed between the transcriptional level of Axin2 and TNFα mRNA in individual mice (Additional file 2: Figure S2.c, Pearson correlation, *r* = 0.845; *p* < 0.001).We also found a significant induction of mRNA levels of transforming growth factor 1 beta (TGFβ1) (Fig. 3f) and Smad7 (Additional file 2: Figure S2d), the negative feedback regulator of TGFβ signaling. Upregulation of hippocampal TGFβ signaling in early stages of EAE is consistent with its CNS specific role in disease initiation [24]. In contrast to the significant increase in microglia number in the acute stage, the number of astrocytes remained unaffected by neuronal injury; however, it was significantly increased in the chronic stage (Fig. 3d). Together these data indicate that the upregulation of *Wnt* activity described above coincides with occurrence of neuronal damage and inflammatory processes at the early stage of disease, whereas in the chronic stage it returned to the basal level.

### *Wnt* signaling promotes neurogenic processes in the DG

**Fig. 4:**
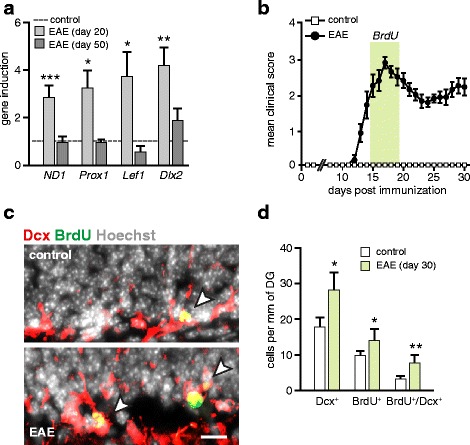
Hippocampal neurogenesis is increased in the acute phase of EAE. **a** Induction of pro-neuronal transcription factors in the hippocampus of mice with passive EAE. qPCR analysis of the *Wnt*-dependent genes (*NeuroD1* (*ND1*), *Prox1*, *Lef1)* and *Dlx2*, a marker of transient amplifying neuroblasts. Histogram represents mean + SEM of fold-changes in gene expression of EAE mice relative to control (PBS) group set as 1. *Gapdh* was used as an endogenous reference. Day 20 (EAE, *n* = 4–8; control, *n* = 4–8); day 50 (EAE, *n* = 4–10; control, *n* = 4–11). Two-tailed, unpaired Student’s *t*-test, * *p* < 0.05, ** *p* < 0.01 and *** *p* < 0.001. **b** Disease course of C57/B6 EAE mice immunized with encephalitogenic MOG_35–55_. Control group (CFA) were injected with an adjuvant cocktail without antigen. Data are shown as a mean clinical score ± SEM. In acute phase of disease (days 15–20; highlighted by green line) EAE and respective control mice received daily i.p. injections of BrdU (50 mg/kg body weight). After a 2-weeks chase period without administration of BrdU mice were sacrificed for histological assessment (day 30). **c** Histological analysis of proliferating cells in the DG of EAE mice. Immunostaining for BrdU (green) and Dcx (red) in hippocampal sections. Arrow heads indicate BrdU^+^/Dcx^+^ co-labeled neuronal progenitors. Nuclei were counterstained with Hoechst (grey). Scale bar, 50 μm. **d** The frequency of BrdU label-retaining cells in the DG is increased in mice with EAE (day 30; *n* = 3 mice; 18–28 sections per mouse) as compared to control mice (*n* = 3 mice; 17–28 sections per mouse). Data is shown as mean + SEM of positive cells per mm of the DG. Two-tailed, unpaired Student’s *t*-test,* *p* < 0.05 and ** *p* < 0.01

In the adult brain, the *Wnt*/β-catenin pathway regulates proliferation and self-renewal of hippocampal stem/progenitor cells [19]. Therefore, we next asked if the increased *Wnt* activity in EAE may trigger proliferation and generation of hippocampal neuronal precursor cells. First, in mice with passively induced EAE, we examined the gene expression of pro-neurogenic transcription factors, known to be regulated by β-catenin/TCF transcriptional activity. We found that the increase of *Wnt* activity during the early stage of disease (day 20) correlates with an upregulation of Neurogenic differentiation 1 (NeuroD1), prospero homeobox 1 (Prox1), distal-less homeobox 2 (Dlx2) and lymphoid enhancer-binding factor 1 (Lef1) mRNA levels (Fig. 4a). Next, we used the BrdU labeling approach to mark dividing progenitors in the hippocampus and to trace the cell fate of newborn cells. Considering the early induction of *Wnt* signaling (Fig. 1c), we administered BrdU at 15 d.p.i. for five consecutive days, followed by a period of 2-weeks without BrdU treatment (Fig. 4b). We found a significant increase in the total number of label-retaining cells in the DG of EAE mice (14 ± 2 cells/mm) in comparison to control mice (10 ± 1 cells/mm). Similarly, the number of newly generated neuroblasts, measured by co-labeling of BrdU^+^-retaining cells with the neuronal progenitor marker Dcx, was increased twofold in EAE mice (8 ± 2 cells/mm in EAE mice; 4 ± 1 cells/mm in control mice) (Fig. 4c-d). Taken together, these results support our previous findings indicating an enhanced *de novo* generation of hippocampal neuronal progenitors in EAE mice [16] and are consistent with the notion that augmented *Wnt* activity in the hippocampus is relevant for activation of neurogenesis in acute disease stages.

### Effects of TNFα on the *Wnt* signaling and hippocampal neurogenesis

Though the induction of *Wnt* signaling was previously reported in the context of various tissue damage paradigms [17, 18], the mechanisms triggering the *Wnt* pathway remain unknown. Therefore, we used the Axin2^lacZ/+^ reporter mice to characterize the possible contribution of particular cytokines, known to be secreted by activated microglia, to promote *Wnt* activity in neural tissue.

**Fig. 5:**
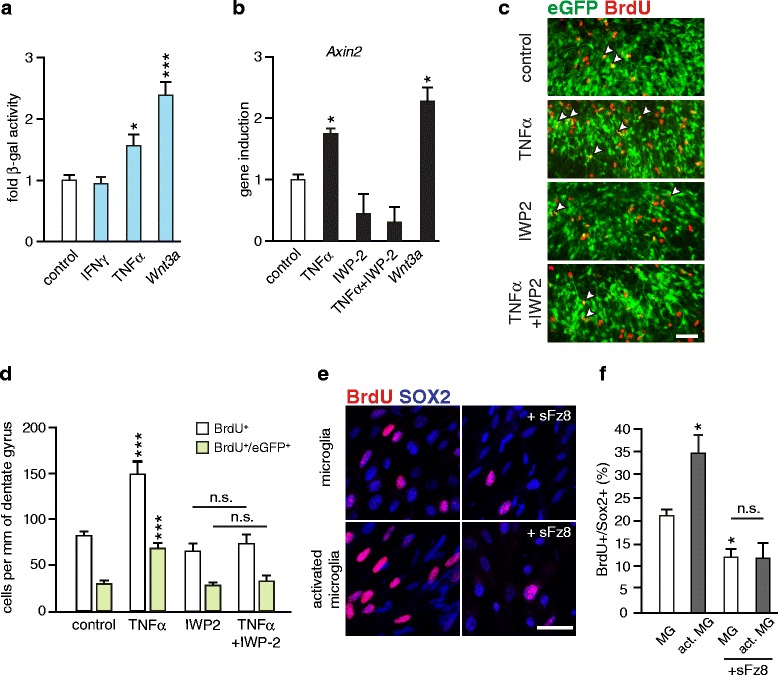
Analysis of *Wnt* activity in organotypic slice cultures (OSCs). **a** β-gal assay. Hippocampal Axin2^lacZ/+^ OSCs were treated for 6 h with *Wnt3a* (20 ng/ml), TNFα (1 ng/ml) or IFNγ (100 U/ml) and the medium was replaced with fresh cytokine-free medium for additional 24 h. Histogram represents mean + SEM of fold-changes of β-gal activity after treatment with *Wnt3a* (*n* = 15), TNFα (*n* = 14) or IFNγ (*n* = 14) relative to controls (PBS, *n* = 20) set as 1. Two-tailed, unpaired Student’s *t*-test, * *p* < 0.05 and *** *p* < 0.001. **b** qPCR analysis of the *Axin2* gene expression. OSCs were treated as described above. IWP-2 (4 μM) was present in the media during the whole experiment. Histogram represents mean + SEM of fold-changes in *Axin2* gene expression after treatment with *Wnt3a* (*n* = 3), TNFα (*n* = 4) or TNFα with IWP-2 (*n* = 3) relative to control (PBS, *n* = 4) OSCs set as 1. *Gapdh* was used as an endogenous reference. Two-tailed, unpaired Student’s *t*-test, * *p* < 0.05. **c**-**d** Histological analysis of proliferating cells in the DG of hippocampal Nestin^eGFP^ OSCs. BrdU was administered to slice culture for 24 h prior to treatment with cytokines (performed as described for Fig. 5a-b). **c** Representative images show the immunostaining for BrdU (red) and eGFP (green). Arrow heads indicate BrdU^+^/eGFP^+^ co-labeled hippocampal progenitors. Scale bar, 50 μm. **d** Inhibition of *Wnts* secretion with IWP-2 (4 μm) abrogates the proliferative effect of TNFα on mitotic activity of hippocampal eGFP^+^ progenitors. Data are shown as mean + SEM of BrdU^+^ or BrdU^+^/eGFP^+^ co-labeled cells per mm of the DG. Control (PBS, *n* = 20 slices); TNFα (*n* = 9 slices), IFNγ (*n* = 11 slices) and *Wnt3a* (*n* = 9 slices); Two-tailed, unpaired Student’s *t*-test, * *p* < 0.05, ** *p* < 0.01 and *** *p* < 0.001. **e**-**f** TNFα - activated hippocampal microglia promotes the proliferation of hippocampal progenitors in vitro. Microglia cultured on transwell inserts were primed by TNFα (1 ng/ml) for 4 h. Transwells were set onto the hippocampal progenitor cultures in proliferating medium containing 5 ng/ml of bFGF in the presence or absence of soluble Frizzled-8 (Fz8; 1 μg/ml). After 24 h dividing cells were labelled with BrdU (2 h) and cells were fixed for immunocytochemistry. **e** Representative images of SOX2^+^ (blue) hippocampal progenitors stained for BrdU (red). Scale bar, 25 μm. **f** Quantification of BrdU-retaining SOX2^+^ cells. Data are shown as mean of four experiments + SEM. Two-tailed, unpaired Student’s *t*-test,* *p* < 0.05

We switched our experimental setup to hippocampal slice cultures, which represent a useful model to study neurogenic processes *ex vivo* [25]. We focused on the role of TNFα and IFNγ, because their expression was significantly induced in the hippocampus of EAE mice (Fig. 3c) and because of independent findings on their potential contribution in tissue regeneration during demyelinating pathology [26–28]. Similar to freshly isolated hippocampal tissue (Fig. 1d), the pattern of *Wnt* activity in cultured slices examined by X-gal staining was mostly confined to the neuronal compartment of the granular layer (Additional file 3: Figure S3a). Treatment with TNFα or *Wnt3a* (used as a positive control [29, 30]) for 6 h and subsequent incubation of Axin2^lacZ/+^ slices in cytokine-free media for 24 h led to a significant upregulation of *Wnt* activity (1.56 ± 0.20 fold and 2.39 ± 0.18 fold, respectively) (Fig. 5a). The increase of *Wnt* signaling was limited to this particular immune cytokine, as the treatment with IFNγ did not have a similar effect. We then investigated whether TNFα may have a direct effect on the induction of *Axin2* in cultured hippocampal progenitor cells. However, the treatment with TNFα was not associated with upregulation of *Axin2* transcription (Additional file 3: Figure S3b). In contrast, *Wnt3a* potently induced *Axin2* gene expression in these cells (Additional file 3: Figure S3b). This result led us to the hypothesis that enhancement of *Wnt* signaling in slice cultures is mediated via an indirect effect of the cytokine on other cell types. To test this, we separated hippocampal astrocytes (GLAST^+^) and microglia (CD11b^+^) from mixed glial cultures using microbeads-based sorting procedure and exposed them to TNFα. Analysis of gene expression of *Wnt* ligands found to be upregulated in acute stage (Fig. 2c) revealed that TNFα significantly induced the expression of *Wnt2*, *Wnt3* and *Wnt5a* in astrocyte cultures and *Wnt3* and *Wnt5a* in microglia cultures (Additional file 3: Figure S3c). We further investigated the effect of inhibition of endogenous *Wnt* ligand secretion on the induction of *Axin2* in hippocampal slice cultures. Treatment with TNFα enhanced *Axin2* expression (Fig. 5b), supporting our results of the β-gal assay (Fig. 5a). Co-treatment with IWP-2 inhibitor, that inactivates porcupine and suppresses the secretion of *Wnt* ligands [31], potently abrogated the positive effect of TNFα (Fig. 5b). Taken together, these data suggest a role of *Wnt* ligands in TNFα-mediated upregulation of hippocampal *Wnt* signaling.

We next asked whether TNFα affects hippocampal neurogenesis and whether *Wnt* ligands contribute to this effect. To assess the proliferation rate of hippocampal progenitors in Nestin^eGFP^ hippocampal slice cultures, we labeled mitotically active cells with BrdU prior to 6 h treatment with the individual cytokines. After a 24-h chase in cytokine-free media the quantification of BrdU-retaining cells was examined. Treatment with TNFα (Fig. 5c-d) or *Wnt3a*, but not IFNγ (Additional file 3: Figure S3d-e), led to a significant increase in the number of BrdU^+^/eGFP^+^ neural progenitor cells. Inhibition of *Wnt* ligand secretion by IWP-2 suppressed the proliferative effect of TNFα (Fig. 5c-d). We further confirmed these results using co-culture experiments. For this approach we used hippocampal microglia because of its prominent activation pattern during the EAE progression (Fig. 3b-c). CD11b-sorted hippocampal microglia cultured on transwell inserts were activated with TNFα for 6 h. After several washing steps, transwells were set onto hippocampal progenitor cultures in proliferating conditions in the presence or absence of soluble Frizzled-8-Fc protein (sFz8). This recombinant protein contains a conservative cysteine-rich region (CRD) and was efficiently used to block *Wnt* ligand binding in hippocampal progenitor cultures [30]. After 24 h, mitotic cells were labeled with BrdU (2 h treatment). TNFα -primed microglia significantly enhanced mitotic activity of SOX2+ progenitor cells (Fig. 5e-f). Blockade of *Wnt* signaling with sFz8 decreased the proliferating rate to the level observed in progenitors co-cultured with non-activated microglia in the presence of sFz8. Taken together, these results are consistent with the observation that TNFα promotes *Wnt* signaling to enhance the initial steps of the hippocampal neurogenic program. In part, this effect is mediated through microglia-dependent *Wnt* ligand production, supporting the pro-neurogenic role of activated microglia in the hippocampus [32–34].

## Discussion

The discovery of *Wnt* pathway activity in the adult CNS in physiological and pathological conditions has raised new questions about the mechanism of its activation. Moreover, it is of therapeutic interest whether the unique involvement of the *Wnt* pathway in developmental processes (such as neurogenesis, oligodendrogenesis, synaptogenesis and axonal guidance) may be useful for facilitation of tissue renewal and repair.

In the present study we made use of Axin2^lacZ/+^ reporter mice to monitor the dynamic changes in hippocampal *Wnt* activity upon acute and chronic stages of EAE, an animal model of MS. Our major finding is that CNS pathology, relevant for acute tissue injury and inflammation, is associated with a transient increase of hippocampal *Wnt* signaling. Enhanced *Wnt/*β-catenin activity was correlated with transient upregulation of canonical *Wnt* ligand (*Wnt2*, *Wnt3*, *Wnt3a*, *Wnt8b* and *Wnt9a*) mRNA levels. We also show that TNFα, an inflammatory cytokine upregulated in the hippocampus of EAE mice (Fig. 3d), can be considered as a potent factor contributing to *Wnt* signaling activation. Furthermore, the induction of *Wnt* activity led to increased proliferation of neuronal progenitors and generation of Dcx^+^ neuronal precursor cells (also known as Type2b). Taken together, our study revealed a link between neuroinflammation and *Wnt*-dependent activation of hippocampal neurogenesis regarding the tissue repair program.

Our finding that activation of *Wnt* signaling is mediated by active inflammatory processes is in line with previous reports on MS pathology. Using different biochemical techniques, the upregulation of *Wnt* ligands (*Wnt2*, *Wnt7a*: microarray data [35]); *Wnt3a*: proteomic analysis [36]) and *Wnt* pathway members such as Axin2 (histological examination [37]), GSK-3β, β-catenin and Tcf4 (proteomic analysis [36]) was demonstrated in active plaques in the white matter and was absent in chronic silent plaques or normal-appearing white matter. In the EAE model, transient induction of *Wnt1* was demonstrated in the subventricular zone (SVZ) of the lateral ventricle [23].

We found that the upregulation of *Wnt*/β-catenin activity in the hippocampus is associated with an induction of several canonical *Wnts* involved in self-renewal and cell fate determination of stem/progenitor cells, synaptic plasticity, axon guidance and tissue homeostasis (reviewed in [38, 39]). Thus, *Wnt3* and *Wnt3a* are potent activators of self-renewal and neuronal fate in the adult hippocampus [19, 29] and cultured neural progenitor cells [30]. *Wnt2* enhances neurogenesis in the developing brain [40] and its induction in the dentate gyrus after electroconvulsive seizures (ECS) [41] was reported to be associated with increased hippocampal neurogenesis [42]. *Wnt8b* regulates dorsal thalamic neurogenesis during zebrafish development [43] and was also shown to enhance the proliferation of cultured rat adult hippocampal progenitors [30]. Expression of *Wnt16a* (human analogue of mouse *Wnt16*) was found in the brain [44]. However, its role in the CNS remains to be examined. Considering the transient induction of *Wnt16* and activation of *Wnt*/β-catenin pathway at the early stage of osteoarthritis [45], it is plausible to assume that *Wnt16* mediates the response to neural tissue injury via promotion of β-catenin signaling. Remarkably, in addition to their role in neural plasticity, *Wnts* likely participate in neuroimmune interactions (reviewed in [46]).

Consistent with previous reports demonstrating the upregulation of different components of canonical *Wnt* pathway in various models of acute spinal cord pathology (such as EAE [22], spinal cord injury [47] and lysolecithin-induced demyelination [37]), we found the enhancement of *Wnt*/β-catenin signaling in the spinal cord of Axin2^lacZ/+^ EAE mice. Notably, in this part of the CNS *Wnt* activity was increased throughout the course of disease and correlated with a large number of inflammatory foci (Additional file 1: Figure S1b), indicating ongoing active inflammatory processes. Thus, it is conceivable that the new waves of inflammation-mediated damage trigger the persistent activation of *Wnt* signaling in the spinal cord.

In line with the idea that acute inflammation enhances *Wnt* signaling, we showed that TNFα is a potent inducer of *Wnt* activity in hippocampal slice cultures. This finding is consistent with previous reports demonstrating the interplay between those two signaling pathways in other cellular contexts [48–51]. Notably, increased proliferation and induction of *Wnt* genes (such as of *Wnt3*, *Wnt5a*, *β-catenin*) was observed upon TNFα-induced trans-differentiation of human mesenchymal stem cells towards the neural lineage [52]. Taking advantage of hippocampal slice cultures as a useful model to study neurogenic processes *ex vivo* [25], we also demonstrated that TNFα-dependent activation of the *Wnt* pathway induces the proliferation of Nestin^+^ progenitor cells in the DG,and this effect is mediated via production of *Wnt* ligands. We further consider hippocampal microglia as the responder cells to TNFα-dependent enhancement of *Wnt* signaling, although other targets of TNFα may also mediate its effect on adult neurogenesis. While the mechanism remains to be elucidated, our data is in line with the role of TNFα-TNFR2 signaling in hippocampal neurogenesis in healthy mice [53] and upon various CNS pathologies. Thus, in the model of status epilepticus, deficiency for TNFR2 drastically abrogates the number of newly generated neurons in the hippocampus [54], though not in the SVZ [55]. Complementary to these findings, infusion of neutralizing antibodies against TNFα had an anti-proliferative effect on hippocampal progenitors in a rat model of stroke [56]. In cultured progenitor cells, TNFα potentially triggers the proliferation of neural stem/progenitor cells [57–59].

Understanding the role of TNFα in MS is complex and has been re-considered in the last years [60, 61]. While anti-TNFα therapy has been used successfully to treat various autoimmune pathologies, it was proven to be deleterious in MS patients [62]. The aggravation of EAE in TNFR2-deficient mice, particularly in the chronic phase [63], may indicate a principal involvement of the TNFα-TNFR2 axis in processes of regeneration and tissue repair. Supporting this idea, recent findings demonstrated the beneficial effect of TNFα/TNFR2 signaling on expansion of the endogenous pool of oligodendrocyte progenitors and subsequent remyelination in a toxin-induced demyelinating model [26].

Activation of neurogenesis was reported in various models of CNS injuries and diseases [64, 65]. In MS brains, immature neurons were found in a subpopulation of subcortical white matter lesions, which may indicate an activation of neurogenic processes serving to compensate neuronal deficits [66]. However, the origin of these cells is unknown. In contrast to this, specification of stem/progenitor cells seems to be shifted towards the glial lineage in the SVZ of MS patients compared to the healthy brain, leading to a diminished generation of Dcx^+^ neuroblasts [67]. Increased proliferation and enhanced generation of immature neurons in the acute stage of EAE was previously reported by our [16] and other groups [68, 69]. Enhancement of neurogenesis in EAE is transient [16, 68] and seems to be specific to the hippocampal neurogenic niche, whereas in the SVZ, the generation of neuroblasts is reduced [23].

Our data raised the possibility that enhancement of *Wnt* activity may trigger hippocampal neurogenesis in order to replenish damaged neurons. Regarding this, it was shown that lentiviral-based overexpression of *Wnt3a* in the striatum enhances neurogenesis and neuronal differentiation, leading to an improved neuronal function in the model of focal ischemic injury [70]. In addition to its neurogenic role, *Wnts* may promote axonal regeneration [71], synaptic transmission [72] and neuronal differentiation [73] in the injured CNS. Prolonged activation of *Wnt* signaling by *Wnt*-activating small molecule potentiator-1 (WASP-1) infusion into the hippocampus improves cognitive function in healthy adult mice and rescues memory loss in a mouse model of Alzheimer’s disease [72]. Our data support these findings, which already raise a considerable interest in *Wnts* regarding the development of novel therapeutic approaches to treat neuronal injury.

## Conclusion

Taken together, our data argue for a role of inflammation in transient activation of hippocampal *Wnt* signaling upon autoimmune mediated injury. Induction of *Wnts* can be accounted as a key factor triggering endogenous repair by engaging the activation of neuronal progenitors in the hippocampal neurogenic niche. Further experiments are needed to test whether therapeutic boosting of *Wnt* activity leads to tissue regeneration and thus provides a strategy to improve hippocampal function.

## Methods

### Mice

The following mice were used for experiments: 6 weeks old female SJL/J mice were purchased from Janvier (*Le Genest-St-Isle, France*). Nestin^eGFP+^ mice [74] were provided by the animal facility of the University of Düsseldorf. In these transgenic mice the second intron enhancer of the rat *nestin* gene was placed upstream of the minimum promoter of *heat shock protein 68* (*HSP68*) fused to eGFP cDNA and a polyadenylation signal. Axin2^lacZ/+^ [75] were received from the European Mouse Mutant Archive (EMMA). In Axin2^lacZ/+^ mice the *lacZ* gene, containing a nuclear localization signal, was introduced in frame to the endogenous *Axin2* (also known as *conductin* or *Axil*) by replacing exon 2 [75]. *Axin2* is a direct target of TCF/LEF1-mediated transcriptional activation that encodes an inhibitor of the *Wnt* signaling pathway. All mice were housed in the animal research facility of the University of Düsseldorf under specific pathogen free conditions, a dark/light cycle of 12 h, a stable temperature of 22–24 °C and unlimited access to food and water.

### EAE induction

All experimental procedures were conducted following the guidelines and protocols approved by the local animal welfare committee (Landesamt für Natur, Umwelt und Verbraucherschutz Nordrhein-Westfalen (LANUV); under protocol numbers G388-11 (active EAE in Axin2^lacZ/+^mice), G197-09 (BrdU injections in EAE mice), G363-09 (passive EAE in SJL/J)) and follow the animal research: reporting of in vivo experiments (*ARRIVE*) criteria and EAE induction guideline [76]. For passive EAE, female SJL/J mice were subcutaneously immunized with 200 μg of recombinant proteolipid protein peptide (PLP_139–151_) (*Pepceuticals*) supplemented with 800 μg heat-inactivated *Mycobacterium tuberculosis* (strain H37RA, *Difco*) emulsified in 200 μl Complete Freund’s Adjuvant (CFA, *Sigma-Aldrich*) per mouse. On day 10 post immunization (p.i.), spleen and lymph node cells were isolated, and the resulting single-cell suspension was re-stimulated with PLP_139–151_ (10 μg/mL) in RPMI 1640 medium supplemented with 10 % fetal calf serum (FCS), penicillin/streptomycin, glutamate, and 2-mercaptoethanol (all from *Invitrogen Life Technologies*). After four days in culture, cells were harvested and injected intraperitoneal (i.p.) into naive female SJL/J mice (3 × 10^7^ cells per mouse). Control mice received a single i.p. injection of PBS. Animals were weighed and scored daily, according to following clinical scale: 0 (no clinical signs), 1 (tail plegia), 2 (abnormal gait), 3 (hind limb paralysis), 4 (complete paralysis) to 5 (death or euthanasia) with intervals of 0.5 [16]. Mice were euthanized either on day 6, 20 or 50 post-transfer. Hippocampi were dissected and stored at −80 °C for total RNA and protein isolation.

To induce active EAE in the Axin2^lacZ/+^ mouse strain, animals were immunized by subcutaneous injection with 200 μg of recombinant myelin oligodendrocyte glycoprotein (MOG_35–55_) peptide (*Pepceuticals*) in CFA (*Sigma-Aldrich*), supplemented with 800 μg heat-inactivated *Mycobacterium tuberculosis* (strain H37RA, *Difco*). Intraperitoneal injections of 200 ng pertussis toxin (PTX, *Sigma-Aldrich*) were performed on the day of immunization and on day 2 [16]. The control group was treated with CFA only and with 200 ng pertussis toxin on day 0 and day 2. Mice were euthanized either on day 20, 30 or 50 post immunization.

### β-Galactosidase assay

Tissue or organotypic slices isolated from Axin2^lacZ/+^ mice were lysed on ice for 15 min in triton-containing lysis buffer (*Applied Biosystems*) and cleared by centrifugation at 10,000 g for 10 min at 4 °C. Then protein concentration was determined by BCA protein assay kit (*Interchim*). 50 μg of protein were applied for measuring β-galactosidase (β-gal) activity in triplicates using the “Galactostar Chemiluminescence Kit” (*Applied Biosystems*) in a white 96-well *NUNC* plate (*Thermo Scientific*) at room temperature (RT) for 2 h using a microplate reader (*TECAN*). The maximum of luminescence intensity for each individual sample was used to calculate mean of triplicate measurements. Lysates of corresponding β-gal-negative brain tissues were used to calculate background intensity.

### Western blotting

Hippocampal tissue isolated from EAE mice was lysed on ice for 15 min in RIPA buffer (50 mM Tris (pH 7.4), 150 mM NaCl, 1 % (v/v) NP40, 0.5 % (v/v) sodium deoxycholate, 0.1 % (v/v) SDS) with protease and phosphatase inhibitors (*Roche*). Lysates were cleared by centrifugation at 14,000 *g* for 20 min at 4 °C and protein concentration was determined using a BCA protein assay Kit (*Interchim*). Protein samples were loaded on 8–16 % gradient “Mini-Protean TGX” gel (*Biorad*). Blotting onto a nitrocellulose membrane was performed using the “Trans-Blot Turbo System” (*Biorad*) for 7 min at 25 Volts. Afterwards, membranes were incubated with blocking buffer (5 % (v/v) skimmed milk in 0.05 % (v/v) PBS/Tween) for 1 h at RT and incubated overnight at 4 °C with following antibodies: mouse anti-neurofilament 160/200 (*Sigma-Aldrich*), mouse anti-PSD-95 (*Abcam*), rabbit anti-β-catenin (*Cell Signalling*), mouse anti-active-β-catenin (ABC, *Cell Signalling*), mouse anti-β-actin (*Sigma-Aldrich*). Primary antibodies were detected by incubation with IR-Dye secondary antibodies (*Li-COR)* for 1 h at RT and subsequently quantified using the Odyssey infrared imaging system (*LI-COR*). Optical density analysis was performed with *ImageJ* software (*NIH*).

### RNA isolation, cDNA synthesis and qPCR

RNA was isolated with a RNA isolation Kit (*Macherey-Nagel*) or Trizol (*Invitrogen Life Technologies*). 1 μg of purified RNA was used for first-strand cDNA synthesis using “Superscript III Reverse Transcriptase” and oligo(dT) in a final volume of 20 μl according to manufacturer’s instruction (*Invitrogen Life Technologies*). Real-time quantification of gene expression was performed using a SYBR Green qPCR assay (*Applied Biosystems*) or TaqMan 5′FAM- and 3′TAMRA-labeled fluorescent probes (*Eurofins MWG*). PCR was performed using an “ABI 7500” real-time PCR system (*Applied Biosystems*) with standard cycling conditions: 40 cycles of 10 s at 95 °C, 60 s at 60 °C. Specificity of the PCR product was confirmed by examination of the dissociation reaction plot and PCR product detection on 2 % agarose gel. The samples were run in duplicates and the level of expression of each gene was normalized to the expression of the housekeeping gene glycerinaldehyd-3-phosphat-dehydrogenase (*GAPDH*). The sequences of all PCR primers used are available on request.

### Organotypic slice cultures

Organotypic slice cultures (OSCs) were generated from 10 days old Axin2^lacZ/+^/nestin^eGFP+^ pups as previously described [77]. Briefly, the hippocampus was cut into 350 μm thick slices using a McIllwain tissue chopper (*GaLa Instrumente*). OSCs were dissociated in ice-cold dissecting medium (Hank’s Balanced Salt Solution (HBSS), *Invitrogen Life Technologies*) complemented with penicillin/streptomycin (100 U/ml, *Invitrogen Life Technologies*), 2.5 mg/ml glucose (*Sigma Aldrich*) and 10 mM kynurenic acid (*Sigma Aldrich*). OSCs were cultured on Millicell-CM culture plate inserts (*Millipore*) in culture medium (50 % (v/v) MEM, 25 % (v/v) HBSS, 25 % (v/v) heat-inactivated horse serum, 2 mM glutamine, penicillin/streptomycin (100 U/ml) (all from *Invitrogen Life Technologies*) and 2.5 mg/ml glucose (*Sigma Aldrich*) for 1 week at 37 °C in a humidified atmosphere with 5 % CO_2_. At this point OSCs were used for further experiments. For analysis of β-gal activity and *Axin2* gene expression hippocampal Axin2^lacZ/+^ OSCs were cultured for seven days followed by treatment with *Wnt3a* (20 ng/ml; *R&D Systems*), interferon gamma (IFNγ, 100 U/ml; Immunotools), TNFα (1 ng/ml; Immunotools) or TNFα (1 ng/ml) with the inhibitor of Porcn function, IWP-2 (4 μM; *R&D Systems*). After 6 h of treatment, the medium was replaced with fresh cytokine-free medium and OSCs were cultured for additional 24 h. Afterwards, tissue was either fixed (β-gal assay; discussed below) or collected for RNA isolation. For the double treatment with IWP-2, the inhibitor was all the time present in the media.

For analysis of neurogenesis in hippocampal OSCs, cultures were first pretreated for 24 h with BrdU (0.5 μM, Sigma) to allow detection of proliferating cells [25]. Next, recombinant cytokines: TNFα (1 ng/ml), interferon gamma (IFNγ, 100 U/ml) (both from *ImmunoTools*) or *Wnt3a* (20 ng/ml, *R&D Systems*) were added to the culture medium for 6 h, followed by washing and replacement with cytokine-free media for additional 24 h. IWP-2 (4 μM, R&D Systems) was added at the same time as the cytokines and was present until the end of the experiment. After this period, slice cultures were directly used to analyse β-gal activity (see above), RNA isolation or fixed with aldehydes for histological examination.

### Histology

Mice were anaesthetized using isoflurane (*Piramal Healthcare*) and perfused with PBS. Brain and spinal cord were dissected and either directly frozen for X-gal staining or postfixed with 4 % (v/v) paraformaldehyde (PFA, *Roth*) for 16 h, followed by dehydration in a 25–30 % (v/v) sucrose solution. Tissue samples were cryopreserved in “TissueTek” (*Sakura Fintek Europe*) at −80 °C. For histological examination brains and spinal cords were cut on a cryostat (*Leica*). 20 μm slices were permeabilized with 0.5 % (v/v) Triton X-100 and blocked in 5 % (v/v) horse serum (*Invitrogen Life Technologies*) and 1 % (v/v) bovine serum albumin in PBS for 2 h. OSCs were washed two times in warm PBS, fixed for 1 h in 4 % PFA and permeabilized for 1 h with 1 % (v/v) Triton X-100 (*Sigma Aldrich*) in PBS. Primary antibodies were diluted in PBS containing 2.5 % (v/v) horse serum with 0.25 % (v/v) Triton X-100 and incubated with tissue samples overnight (or 48 h for OSCs) at 4 °C. Following primary antibodies were used: guinea pig anti-GFAP (1:1000; Synaptic Systems), rat anti-CD68 (1:350; Biolegend), goat anti-SOX2 (1:250; Santa Cruz); mouse anti-iNOS (1:100; BD Transduction Laboratories), rabbit anti-Iba1 (1:500, *Wako Pure Chemical Industries*), rabbit anti-GFP (1:1000, *Abcam*) goat anti-Doublecortin (Dcx, 1:250, Santa Cruz Biotech) and Hoechst 33342 dye (*Molecular Probes*). Visualization was performed by incubation with fluorescent Cy2-, Cy3- and Cy5-conjugated secondary antibodies (*Millipore*) for 1–2 h at RT. Hoechst dye 33258 (*Molecular Probes*) was used to counterstain nuclei followed by mounting on glass slides with “Immuno Mount” (*Thermo Scientific*). Immunostainings were analyzed on an *Olympus* BX51 microscope and overlaid using *Photoshop 13.0* software (*Adobe*).

Quantification of Iba1^+^ and GFAP^+^ cells in the dentate gyrus has been performed in four animals per group. The number of positive cells per animal was calculated as a mean of labeled cells in mm^2^ of the DG area in 3–4 sections (200 μm distance between sections). The ratio of micron per pixel (1 pixel = 0.16125 μm at magnification x 40) was used to calculate the area analyzed.

For X-gal staining, 20 μm thick cryoslices or OSCs, prepared from Axin2^lacZ/+^ mice, were fixed with PBS containing 2 % (v/v) formaldehyde, 0.2 % (v/v) glutaraldehyde and 0.02 % (v/v) NP40 (all from *Sigma Aldrich*) for 5 min. After washing, slices were incubated for 2 h at 37 °C in X-gal staining solution, containing 10 mM K_3_Fe(CN)_6_, 10 mM K_4_Fe(CN)_6_, 0.02 % (v/v) NP40, 2 mM MgCl_2_ and X-gal (0.5 mg/ml) (all from *Sigma Aldrich*) dissolved in PBS. Samples were fixed with 4 % (v/v) PFA for 15 min and mounted on glass slides with “Immuno Mount” (*Thermo Scientific*).

### BrdU treatment and immunostaining

For labeling of proliferating cells, 50 mg/kg body weight of 5-bromo-20-desoxyuridine (BrdU; *Sigma Aldrich*) was injected i.p. daily for five following days starting at the peak of disease (14 d.p.i.). EAE animals and healthy controls were euthanized and perfused 2 weeks after the first BrdU-injection. Mouse hippocampi were collected and cryopreserved (see histology part) and afterwards processed as previously described [16]. Briefly, sections were incubated in 2 N HCl for 30 min at 37 °C and rinsed with 100 mM tetraborate buffer. Sections were incubated with primary rat anti-BrdU antibody (1:400; *Upstate*) and goat anti-Dcx antibody (1:500; Santa Cruz Biotechnology) overnight at 4 °C, followed by incubation with appropriate Cy2/Cy3-conjugated secondary antibodies (Millipore) for 1 h at RT.

### Primary hippocampal cell cultures

#### Tissue processing and preparation of mixed glial cell population

Whole hippocampus from 2 to 5-day-old C57BL/6 mice were dissected and washed with ice cold HBSS supplemented with 0.2 % glucose. Tissue was finely minced with a razor blade and digested by StemPro Accutase (Gibco) for 20 min at 37 °C followed by 10 min digestion with 0.05 % Trypsin-EDTA (Gibco), DNase (250 U/ml, Worthington). Tissue was rinsed with DMEM supplemented with 10 % heat-inactivated fetal calve serum (FCS; Gibco), gently triturated by passing through a 1-ml serological pipette until a homogenous suspension appeared and filtered through a 40 μm cell strainer (BD Biosciences). Cells were suspended in Neurobasal medium supplemented with B27, glutamax, 1 % penicillin/streptomycin (all from Gibco) (hereinafter referred to as NB media).

#### Microglia and astrocyte cell cultures

Isolated hippocampal cells were plated on poly-ornithine-coated petri dishes and cultured in 10 ml of NB media (see above) supplemented with 10 % (v/v) FCS and 20 ng/ml of macrophage colony stimulating factor (M-CSF; Immunotools). 50 % of culture media was replaced every 4 days with fresh media. After 2–3 weeks incubation in a 5 % CO_2_ incubator at 37 °C, cells were collected by dissociation with StemPro Accutase (Gibco) for positive selection of CD11b^+^ cells or GLAST^+^ cells using magnetic microbeads (Miltenyi Biotech), according to the manufacturer’s instructions. This approach results in about 90 % purity of primary microglia cells and 95 % purity of primary astrocytes determined by immunostaining for Iba-1 and GFAP, respectively. Microglia and astrocytes were harvested, washed and plated on poly-L-ornitine coated plates or coverslips at densities appropriate for each assay.

#### Hippocampal progenitor cultures

Isolated hippocampal cells were cultured in serum-free proliferating conditions on uncoated Petri plates in NB supplemented with 20 ng/ml bFGF (Immunotools). After 3–4 days divided cells formed free-floating neurospheres. bFGF was added every second day. 30 % of culture media was replaced every 4 days with fresh media. After 1–2 weeks in culture neurospheres were collected and cells were dissociated by trypsin-EDTA solution. For further analysis, cells were cultured as adherent cells in proliferating medium on poly-ornithine-coated petri dishes or glasses. For gene expression analysis cells were treated for 6 h with mouse recombinant TNFα (0.2 ng/ml or 1 ng/ml; R&D) or *Wnt3a* (20 ng/ml; R&D).

#### Coculture of hippocampal progenitors with microglia

2 × 10^5^ CD11b^+^ cells in 200 μl NB media were added to the upper well of each Transwell^tm^ inserts (0.2 μm diameter holes, Ibidi) for 24 h. In parallel, 4 × 10^4^ hippocampal progenitor cells dissociated from neurospheres were cultured separately on poly-L-ornithine coated coverslips in proliferating NB media containing 20 ng/ml bFGF. 24 h after culturing of microglia on transwells, cells were activated with TNFα (1 ng/ml) for 4 h and washed carefully with NB media. After this, the transwells containing microglia were set on hippocampal progenitor cultures and incubated for 24 h in proliferating NB media (containing 5 ng/ml bFGF) in the presence or absence of soluble Frizzled-8 (sFz8; 1 μg/ml; R&D). 10 μg/ml of BrdU was added for last 2 h of experiment to allow detection of proliferating cells. Goat anti-SOX2 (1:250; Santa Cruz) and rat anti-BrdU (1:500; Upstate) primary antibody were used to detect mitotically active cells in hippocampal progenitor cultures.

### Statistical Analysis

All values in the figures are shown as mean + SEM. Statistical analysis was performed with GraphPad Prism 5 (GraphPad Software). The p values of *p < 0.05; **p < 0.01; ***p < 0.001 were determined to be statistically significant by using Student’s *t*-test.

## Abbreviations

ABC, active-β-catenin; ARRIVE, Animal Research: Reporting of In Vivo Experiments; BrdU, 5-Bromo-20-desoxyuridine; CFA, Complete Freund’s Adjuvant; CNS, central nervous system; d.p.i., days post immunization; Dcx, doublecortin; DG, dentate gyrus; EAE, experimental autoimmune encephalomyelitis; ECS, electroconvulsive seizures; FCS, fetal calf serum; GAPDH, glycerinaldehyd-3-phosphat dehydrogenase; GSK-3β, glycogensynthase-kinase-3 beta; HBSS, hank’s balanced salt solution; HSP68, heat shock protein 68; i.p., intraperitoneal; IFNγ, interferon gamma; IL-1β, interleukin-1 beta; LANUV, Landesamt für Natur, Umwelt und Verbraucherschutz Nordrhein-Westfalen; Lef1, lymphoid enhancer-binding factor 1; LPC, lysolecithin; MOG, myelin oligodendrocytes glycoprotein; MS, multiple sclerosis; NeuroD1, neurogenic differentiation 1; NF-H, neurofilament-H; OSC, organotypic slice culture; PFA, paraformaldehyde; PLP, proteolipid protein peptide; Prox1, prospero homeobox 1; PSD-95, postsynaptic protein 95; PTX, pertussis toxin; RT, room temperature; SEM, standard error of mean; SGZ, subgranular zone; SVZ, subventricular zone; TCF, transcription factor; TGFβ1, transforming growth factor beta 1; TNFα, Tumor necrosis factor alpha; WASP-1, *Wnt*-activating small molecule potentiator-1.

## Additional files

Additional file 1: Figure S1. Upregulation of *Wnt *activity in the hippocampus and the spinal cord correlates with disease severity. (a) Iba1 immunostaining (red) of spinal cord sections from Axin2^lacZ/+^ EAE mice at day 20. The white dashed line highlights lesion areas; white matter tissue is outlined by yellow dashed line. Nuclei were counterstained with Hoechst (blue). Scale bar, 100 μm. (b) Histogram represents the average number of lesions per section of each EAE time point as mean + SEM. Spinal cord: day 20 (n = 4; 15–35 sections/mouse), day 30 (n = 3; 10–23 sections/mouse), day 50 (n = 3; 10–17 sections/mouse). (c) Representative image of lesion site in the DG. Iba1 (red) immunostaining in hippocampal section from EAE mouse at day 30. Nuclei (grey). Scale bar, 50 μm. (d) Representative image of neural stem cells located in the SGZ. SOX2 (green) and GFAP (red). Arrowheads indicate SOX2^+^/GFAP^+^ radial glia-like cells and arrows indicate SOX2^+^/GFAP^+^ horizontal progenitors. Nuclei (grey). Scale bar, 25 μm. (e+f) β-gal assay of cortical and cerebellar EAE tissue revealed no changes in *Wnt* activity. Histograms represent mean + SEM of fold-changes of β-gal activity in EAE mice relative to controls (CFA) set as 1. Day 20 (EAE, n = 6; CFA, n = 3); day 30 (EAE, n = 4; CFA, n = 3); day 50 (EAE, n = 8; CFA, n = 4). (g) qPCR analysis of *Axin2* in different CNS parts in acute EAE (day 20). Data represent mean of fold-changes + SEM of gene expression in EAE mice (n = 6) relative to controls (CFA; n = 6) set as 1. Expression of *Axin2* was normalized to *Gapdh.* (d) Regression analysis shows correlation between β-gal activity in inflamed CNS tissues and clinical disease score in EAE animals. Pearson correlation, r = 0.58; p = 0.018 (hippocampus) and r = 0.52, p = 0.036 (spinal cord). Statistics: Two-tailed, unpaired Student’s t-test,* * p < 0.05 and ** p < 0.01.  (PDF 6160 kb) Additional file 2: Figure S2. Gene expression analysis in the hippocampal tissue of EAE mice. (a) qPCR analysis of selected *Wnt* ligands in the hippocampus at early (day 20) and chronic (day 50) stages of passive EAE. Histogram represents mean + SEM of fold-changes relative to control group (PBS) set as 1. Gene expression was normalized to *Gapdh.* Day 20 (EAE, *n* = 4–6; control, *n* = 4); day 50 (EAE, *n* = 8–13; control, *n* = 7–11). (b) qPCR analysis of selected *Wnt* ligands in the cortex, cerebellum and spinal cord at early stages of passive EAE (day 20). Histogram represents mean + SEM of fold-changes relative to control group (PBS) set as 1. Gene expression was normalized to *Gapdh.* Six control and six EAE mice were analysed for each part of the CNS. n.d. not detectable. (c) A regression analysis shows correlation between the *TNFα* and the *Axin2* gene expression levels examined in hippocampal tissue of individual EAE animals. Pearson correlation, *r* = 0.8458; *p* < 0. 001. (d) qPCR analysis shows transient upregulation of *Smad7* expression in the hippocampus of mice with passively transferred EAE. Data represent mean + SEM of fold-changes of gene expression in EAE mice relative to respective controls (PBS) set as 1. Expression was normalized to *Gapdh*. Day 20 (EAE, *n* = 4; control, *n* = 4); day 50 (EAE, *n* = 4; control, *n* = 4). Two-tailed, unpaired Student’s *t*-test, * *p* < 0.05 and ** *p* < 0.01. (PDF 430 kb) Additional file 3: Figure S3. Effect of TNFα on gene expression of *Axin2* and *Wnt* ligands in hippocampal cells. (a) The distribution pattern of LacZ expression in Axin2^lacZ/+^ hippocampal slice cultures. X-gal staining of hippocampal slices (postnatal day 10) shows LacZ expression predominantly in the granular cell layer (gcl).  Scale bar, 50 μm. (b) qPCR analysis of *Axin2, Wnt3*, *Wnt8b* and *Wnt5a* gene expression in hippocampal progenitors after 6 h treatment with TNFα (0.2 ng/ml or 1.0 ng/ml; n = 3) or *Wnt3a* (20 ng/ml; n = 3). Histogram represents mean + SEM of fold-changes of gene expression relative to controls (PBS, n = 3) set as 1. (c) qPCR analysis of gene expression of selected *Wnt* ligands in hippocampal astrocytes (GLAST+) and microglia (CD11b+) cells treated with TNFα (0.2 ng/ml or 1.0 ng/ml; n = 3) for 6 h. Histogram represents mean + SEM of fold-changes of gene expression relative to controls (PBS, n = 3) set as 1. (c) Histological analysis of proliferating cells in the DG of hippocampal Nestin^eGFP^ OSCs. Hippocampal slices were cultured for seven days followed by 6 h treatment with *Wnt3a* (20 ng/ml) or IFNγ (100 U/ml) and additional incubation in cytokine-free medium for 24 h. BrdU was administered for 24 h prior to treatment with cytokines. Representative images show BrdU (red) and eGFP (green). Arrow heads indicate BrdU^+^/eGFP^+^ co-labeled hippocampal progenitors. Scale bar, 50 μm. (d) The frequency of BrdU label-retaining cells in the DG of hippocampal Nestin^eGFP^ OSCs is increased after treatment with Wnt3a. Data are shown as mean + SEM of cells per mm of the DG. Control (PBS, n = 15–20 slices); IFNγ (n = 4–11 slices) and *Wnt3a* (n = 8–14 slices). Statistics: Two-tailed, unpaired Student’s t-test,* * *p* < 0.05, ** *p* < 0.01 and *** *p* < 0.001.  (PDF 1448 kb)
