# Supplementary figures and images for: Activation of Wnt signaling promotes hippocampal neurogenesis in experimental autoimmune encephalomyelitis
Source: Mol Neurodegener. 2016 Jul 14;11:53. doi: 10.1186/s13024-016-0117-0 (PMC4969720; doi:10.1186/s13024-016-0117-0)

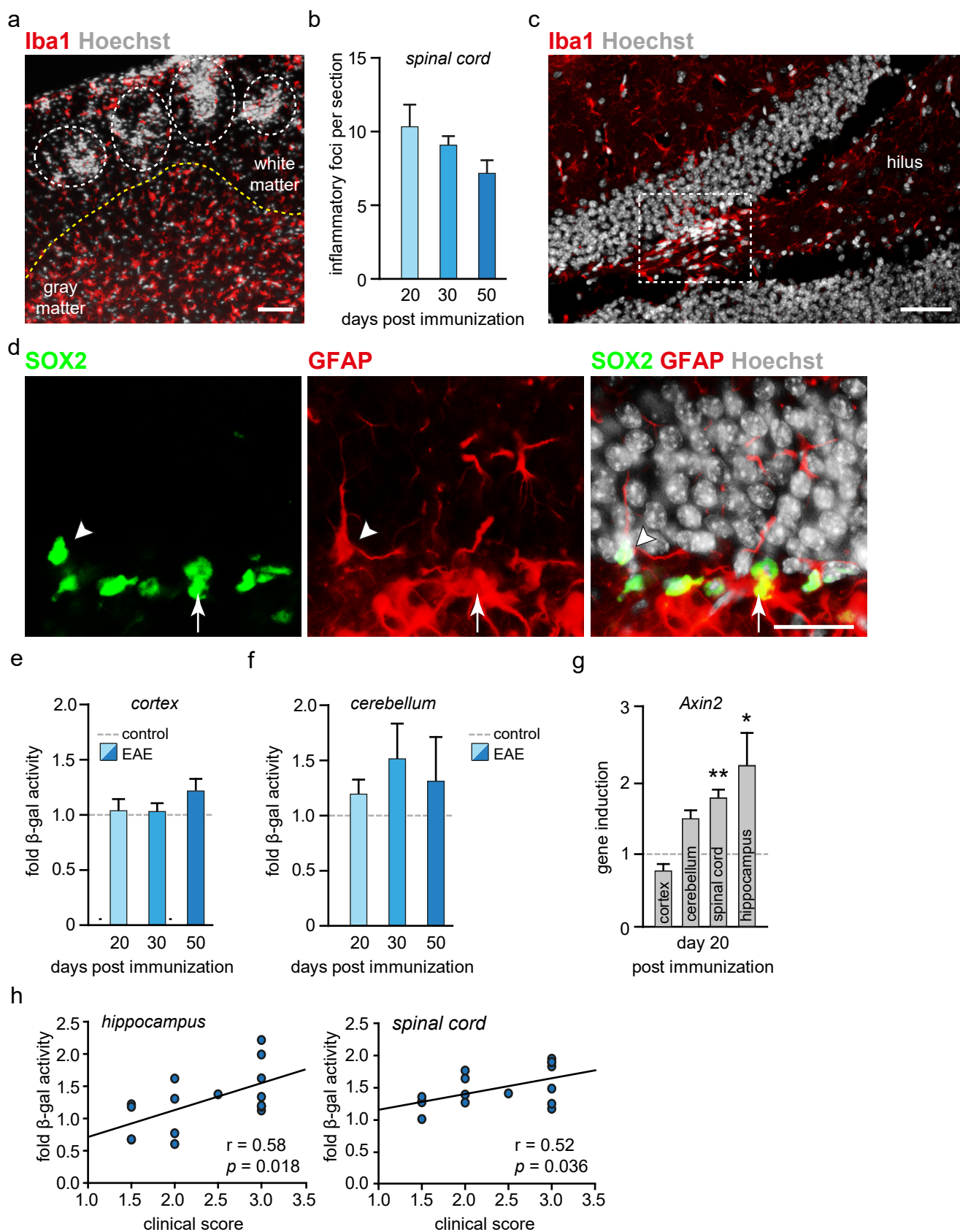

Supplement: Additional file 1: Figure S1. — Upregulation of Wnt activity in the hippocampus and the spinal cord correlates with disease severity. (a) Iba1 immunostaining (red) of spinal cord sections from Axin2lacZ/+ EAE mice at day 20. The white dashed line highlights lesion areas; white matter tissue is outlined by yellow dashed line. Nuclei were counterstained with Hoechst (blue). Scale bar, 100 μm. (b) Histogram represents the average number of lesions per section of each EAE time point as mean + SEM. Spinal cord: day 20 (n = 4; 15–35 sections/mouse), day 30 (n = 3; 10–23 sections/mouse), day 50 (n = 3; 10–17 sections/mouse). (c) Representative image of lesion site in the DG. Iba1 (red) immunostaining in hippocampal section from EAE mouse at day 30. Nuclei (grey). Scale bar, 50 μm. (d) Representative image of neural stem cells located in the SGZ. SOX2 (green) and GFAP (red). Arrowheads indicate SOX2+/GFAP+ radial glia-like cells and arrows indicate SOX2+/GFAP+ horizontal progenitors. Nuclei (grey). Scale bar, 25 μm. (e+f) β-gal assay of cortical and cerebellar EAE tissue revealed no changes in Wnt activity. Histograms represent mean + SEM of fold-changes of β-gal activity in EAE mice relative to controls (CFA) set as 1. Day 20 (EAE, n = 6; CFA, n = 3); day 30 (EAE, n = 4; CFA, n = 3); day 50 (EAE, n = 8; CFA, n = 4). (g) qPCR analysis of Axin2 in different CNS parts in acute EAE (day 20). Data represent mean of fold-changes + SEM of gene expression in EAE mice (n = 6) relative to controls (CFA; n = 6) set as 1. Expression of Axin2 was normalized to Gapdh. (d) Regression analysis shows correlation between β-gal activity in inflamed CNS tissues and clinical disease score in EAE animals. Pearson correlation, r = 0.58; p = 0.018 (hippocampus) and r = 0.52, p = 0.036 (spinal cord). Statistics: Two-tailed, unpaired Student’s t-test,* * p < 0.05 and ** p < 0.01. (PDF 6160 kb) [file 13024_2016_117_MOESM1_ESM.pdf]

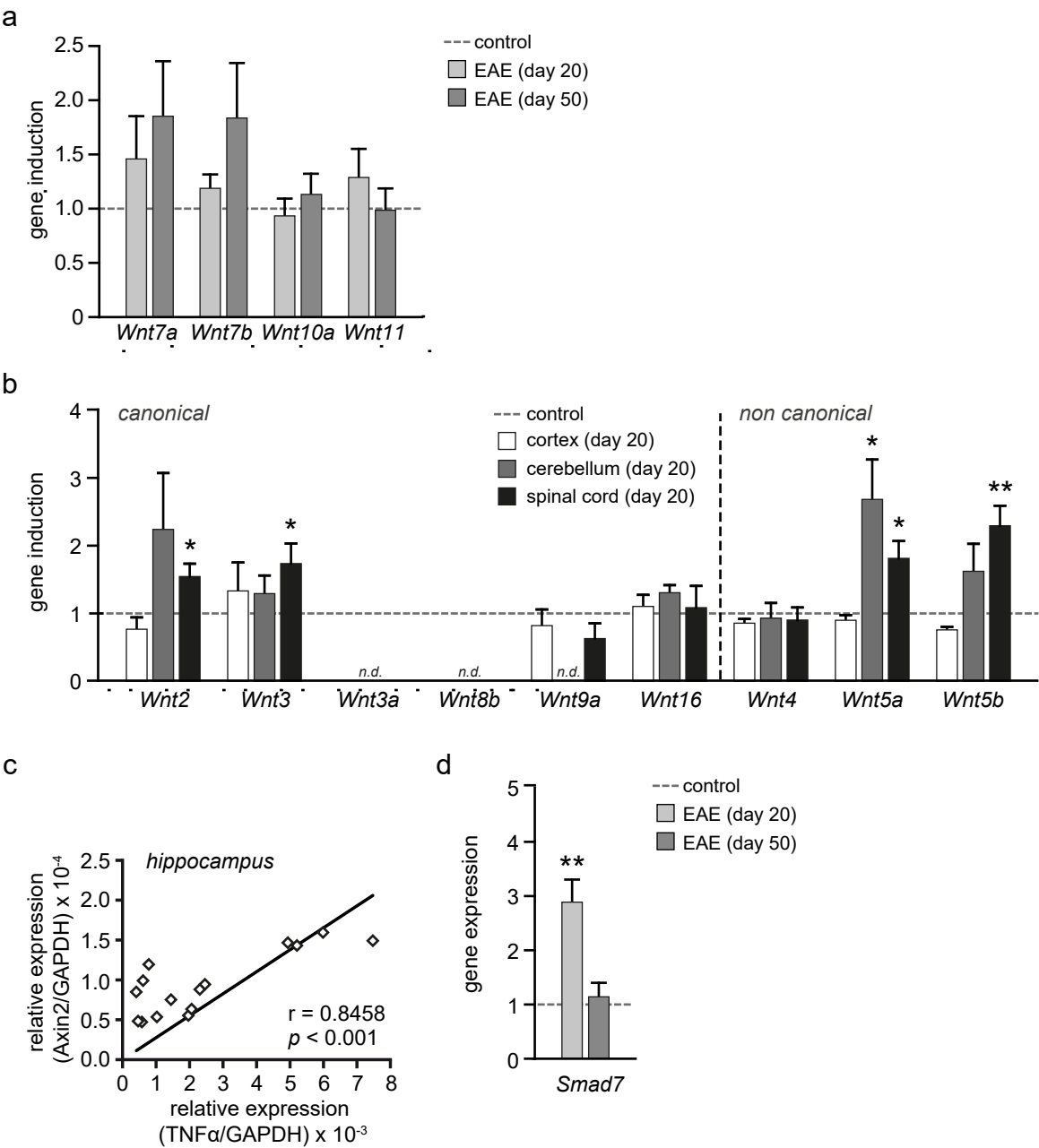

Supplement: Additional file 2: Figure S2. — Gene expression analysis in the hippocampal tissue of EAE mice. (a) qPCR analysis of selected Wnt ligands in the hippocampus at early (day 20) and chronic (day 50) stages of passive EAE. Histogram represents mean + SEM of fold-changes relative to control group (PBS) set as 1. Gene expression was normalized to Gapdh. Day 20 (EAE, n = 4–6; control, n = 4); day 50 (EAE, n = 8–13; control, n = 7–11). (b) qPCR analysis of selected Wnt ligands in the cortex, cerebellum and spinal cord at early stages of passive EAE (day 20). Histogram represents mean + SEM of fold-changes relative to control group (PBS) set as 1. Gene expression was normalized to Gapdh. Six control and six EAE mice were analysed for each part of the CNS. n.d. not detectable. (c) A regression analysis shows correlation between the TNFα and the Axin2 gene expression levels examined in hippocampal tissue of individual EAE animals. Pearson correlation, r = 0.8458; p < 0. 001. (d) qPCR analysis shows transient upregulation of Smad7 expression in the hippocampus of mice with passively transferred EAE. Data represent mean + SEM of fold-changes of gene expression in EAE mice relative to respective controls (PBS) set as 1. Expression was normalized to Gapdh. Day 20 (EAE, n = 4; control, n = 4); day 50 (EAE, n = 4; control, n = 4). Two-tailed, unpaired Student’s t-test, * p < 0.05 and ** p < 0.01. (PDF 430 kb) [file 13024_2016_117_MOESM2_ESM.pdf]

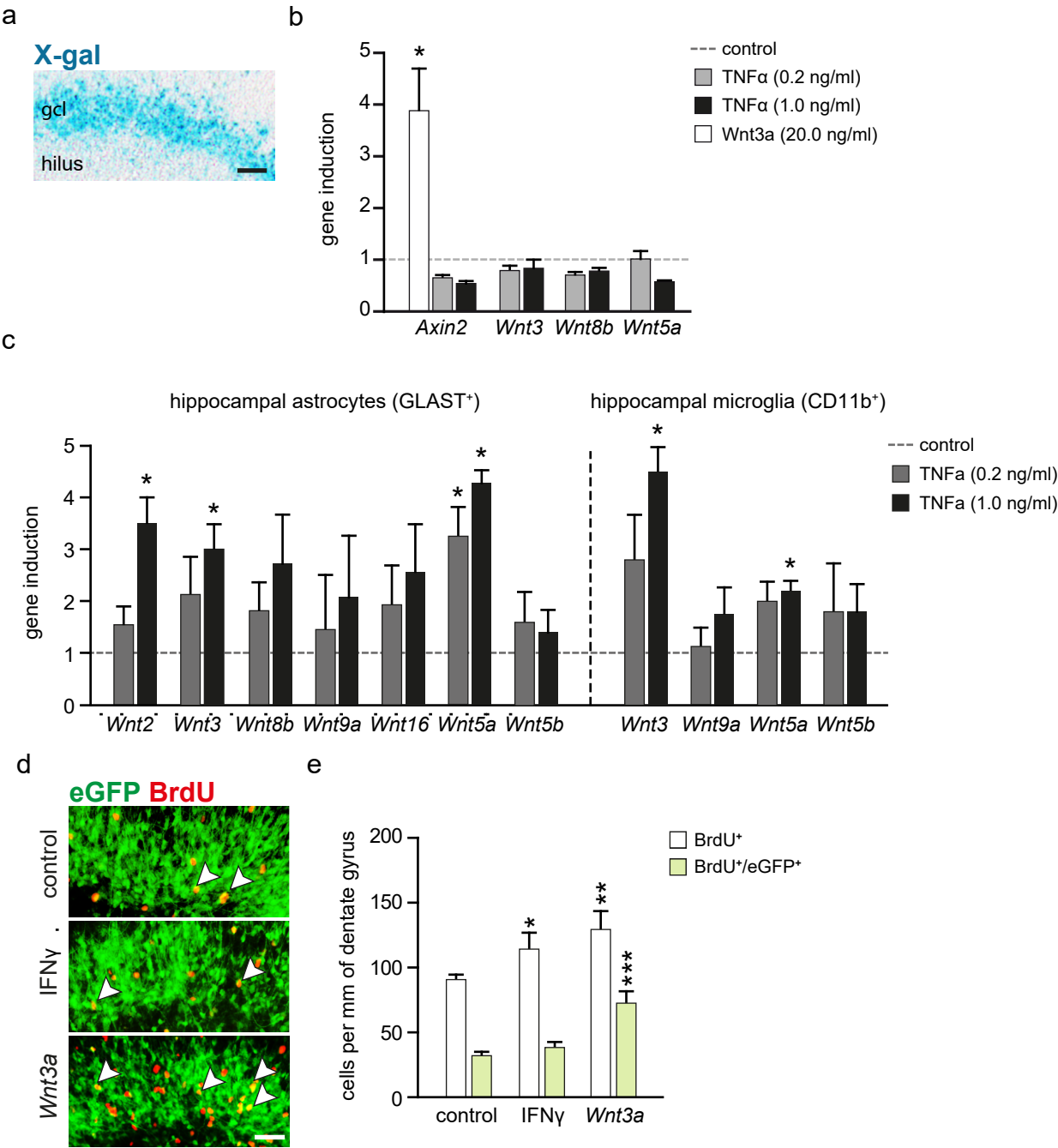

Supplement: Additional file 3: Figure S3. — Effect of TNFα on gene expression of Axin2 and Wnt ligands in hippocampal cells. (a) The distribution pattern of LacZ expression in Axin2lacZ/+ hippocampal slice cultures. X-gal staining of hippocampal slices (postnatal day 10) shows LacZ expression predominantly in the granular cell layer (gcl). Scale bar, 50 μm. (b) qPCR analysis of Axin2, Wnt3, Wnt8b and Wnt5a gene expression in hippocampal progenitors after 6 h treatment with TNFα (0.2 ng/ml or 1.0 ng/ml; n = 3) or Wnt3a (20 ng/ml; n = 3). Histogram represents mean + SEM of fold-changes of gene expression relative to controls (PBS, n = 3) set as 1. (c) qPCR analysis of gene expression of selected Wnt ligands in hippocampal astrocytes (GLAST+) and microglia (CD11b+) cells treated with TNFα (0.2 ng/ml or 1.0 ng/ml; n = 3) for 6 h. Histogram represents mean + SEM of fold-changes of gene expression relative to controls (PBS, n = 3) set as 1. (c) Histological analysis of proliferating cells in the DG of hippocampal NestineGFP OSCs. Hippocampal slices were cultured for seven days followed by 6 h treatment with Wnt3a (20 ng/ml) or IFNγ (100 U/ml) and additional incubation in cytokine-free medium for 24 h. BrdU was administered for 24 h prior to treatment with cytokines. Representative images show BrdU (red) and eGFP (green). Arrow heads indicate BrdU+/eGFP+ co-labeled hippocampal progenitors. Scale bar, 50 μm. (d) The frequency of BrdU label-retaining cells in the DG of hippocampal NestineGFP OSCs is increased after treatment with Wnt3a. Data are shown as mean + SEM of cells per mm of the DG. Control (PBS, n = 15–20 slices); IFNγ (n = 4–11 slices) and Wnt3a (n = 8–14 slices). Statistics: Two-tailed, unpaired Student’s t-test,* * p < 0.05, ** p < 0.01 and *** p < 0.001. (PDF 1448 kb) [file 13024_2016_117_MOESM3_ESM.pdf]
